# Supplementary figures and images for: Profiling G protein-coupled receptors of Fasciola hepatica identifies orphan rhodopsins unique to phylum Platyhelminthes
Source: Int J Parasitol Drugs Drug Resist. 2018 Feb 5;8(1):87–103. doi: 10.1016/j.ijpddr.2018.01.001 (PMC6114109; doi:10.1016/j.ijpddr.2018.01.001)

A

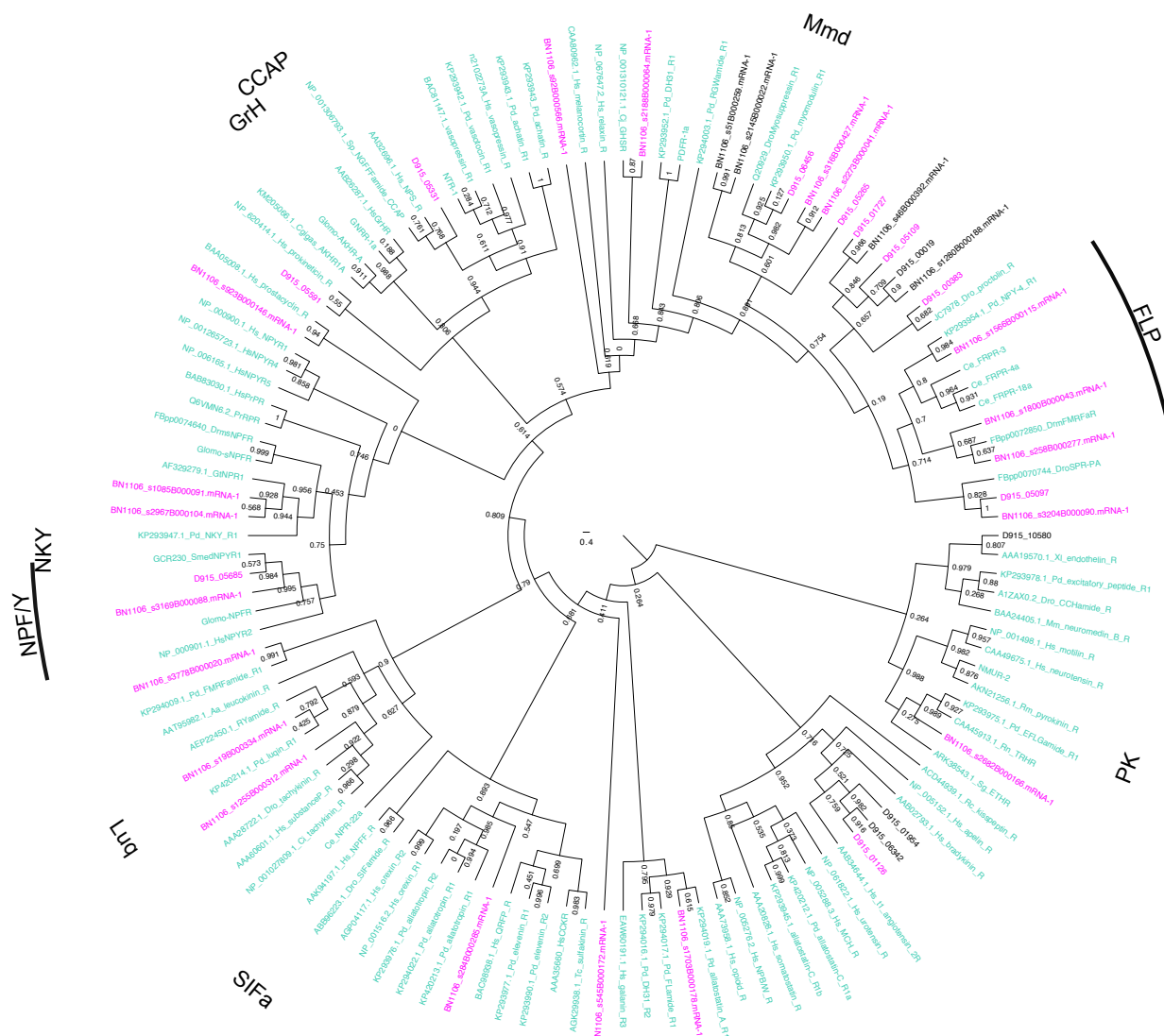

B

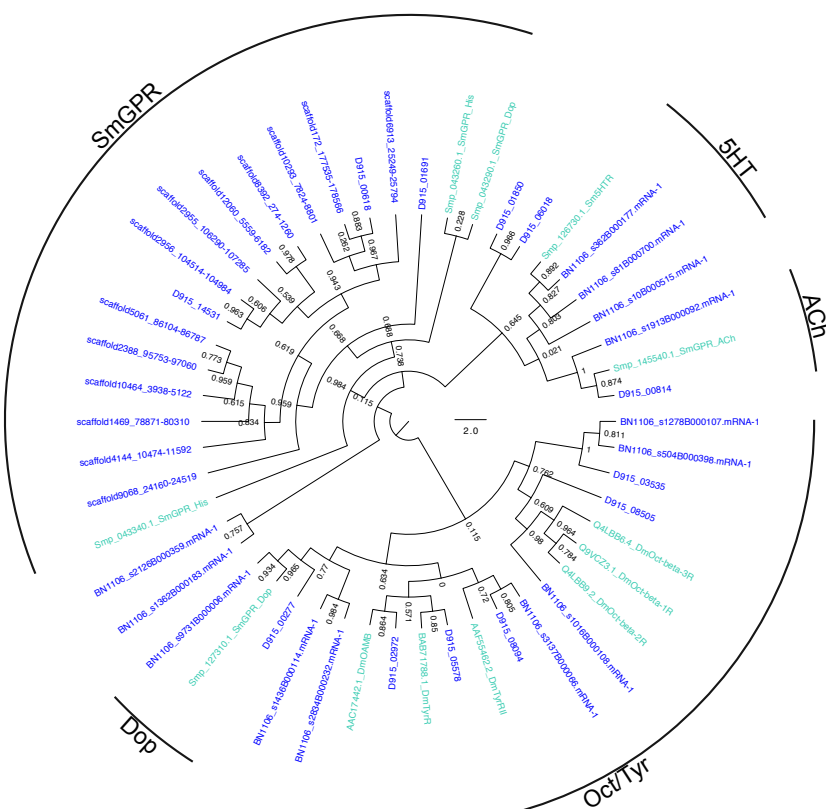

Supplement: S5 Fig — Phylogenetic comparison of Fasciola hepatica GPCRs with deorphanised bilaterian GPCRs. (A) Peptide receptors (F. hepatica black or magenta as described in Fig x); (B) Amine receptors (F. hepatica dark blue); in all cases, non-flatworm receptors are coloured light blue. In (A) outer labels indicate positions of receptors for neuropeptide families previously reported in flatworms (McVeigh et al., 2009, Collins et al., 2010, Koziol et al., 2016); in (B), outer labels represent major groups containing phylogenetically similar F. hepatica sequences. Trees were midpoint rooted, maximum likelihood phylogenies of transmembrane domains I-VII. Numbers at nodes indicate statistical support from approximate likelihood ratio test (aLRT). Scale bars at the centre of each tree indicate number of substitutions per site. Abbreviations: ACh, acetylcholine; CCAP, crustacean cardioactive peptide; Dop, dopamine; FLP, FMRFamide-like peptide; GrH, gonadotropin-releasing hormone; Luq, luqin; Mmd, myomodulin; NKY, neuropeptide KY; NPF/Y, neuropeptide F/Y; Oct, octopamine; PK, pyrokinin; SIFa, SIFamide; Tyr, tyramine; SmGPR, schistosome GPCRs; 5HT, 5-hydroxytryptamine. [file mmc5.pdf]

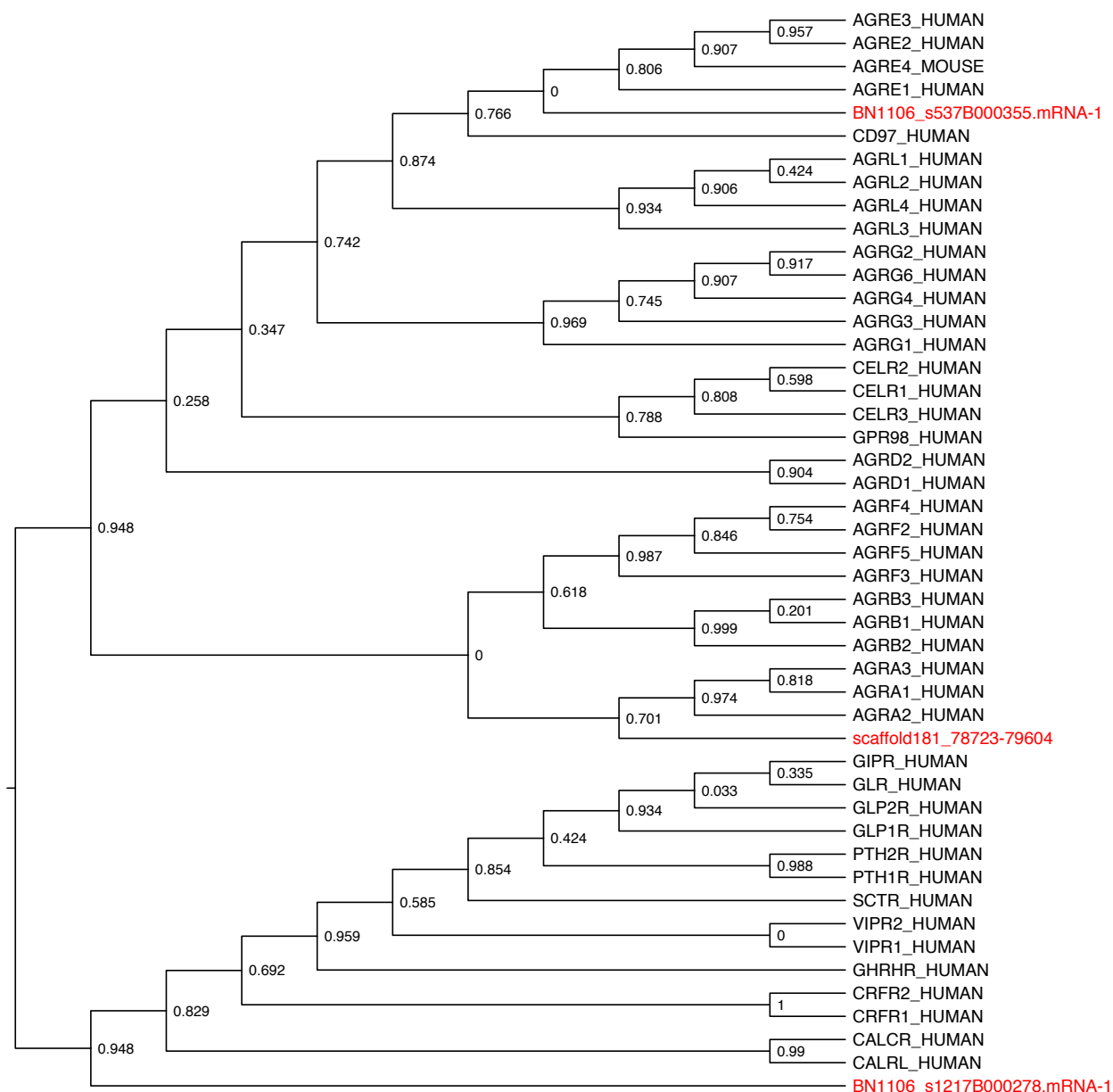

0.3

Supplement: S7 Figure — Class B receptor phylogeny. Maximum likelihood phylogeny of class B (adhesion and secretin) GPCRs from Fasciola hepatica (red) and human (black). Dataset supports designation of two adhesion and one secretin GPCRs in F. hepatica. Tree was a midpoint rooted, maximum likelihood phylogeny of transmembrane domains I-VII. Numbers at nodes indicate statistical support from approximate likelihood ratio test (aLRT). Scale bars at the centre of each tree indicate number of substitutions per site. [file mmc7.pdf]
